# Effects of Aromatherapy on Chemotherapy-Induced Gastrointestinal Toxicity in Patients With Cancer: Protocol for a Systematic Review and Meta-Analysis of Randomized Controlled Trials

**DOI:** 10.2196/76350

**Published:** 2025-12-12

**Authors:** Hongrui Shi, Xin Chen, Xinxin Fan, Mengqi Liu, Wei Peng, Wei Zou

**Affiliations:** 1 College of Nursing, Shanxi University of Chinese Medicine, Jinzhong, China; 2 Department of Oncology, West China School of Public Health and West China Fourth Hospital, Sichuan University, No.18, Section 3, Renmin South Road, Chengdu, 610041, China, +86-28-85502628; 3 Department of Urology/Pelvic Floor and Andrology, West China School of Public Health and West China Fourth Hospital, Sichuan University, Chengdu, China; 4 Research Center for Palliative Care, West China-PUMC C.C. Chen Institute of Health, Sichuan University, Chengdu, China

**Keywords:** aromatherapy, chemotherapy, gastrointestinal toxicity, cancer, protocol, systematic review and meta-analysis

## Abstract

**Background:**

Chemotherapy-induced gastrointestinal toxicity (CIGT) is a common and distressing adverse effect in cancer care, manifesting as nausea, vomiting, appetite loss, oral mucositis, constipation, and diarrhea. These symptoms severely impair patients’ quality of life, reduce treatment adherence, and may lead to premature therapy discontinuation. Aromatherapy, a complementary therapy using plant-derived essential oils, has shown potential benefits for alleviating CIGT symptoms; however, most existing systematic reviews focus solely on nausea and vomiting, leaving its effects on other CIGT symptoms under-studied. Moreover, the influence of essential oil types, intervention forms, and intervention durations on therapeutic outcomes remains unclear.

**Objective:**

This systematic review and meta-analysis aims to comprehensively evaluate the efficacy and safety of aromatherapy for the full spectrum of CIGT symptoms in patients with cancer and to clarify how essential oil types, intervention forms, and intervention durations influence treatment outcomes.

**Methods:**

Nine databases (PubMed, Cochrane Library, Web of Science, Embase, Cumulative Index to Nursing and Allied Health Literature, Chinese National Knowledge Infrastructure, Wanfang, Chinese Science and Technology Journal Database, and SinoMed); the World Health Organization (WHO) Trials Portal; and the Chinese Clinical Trial Registry will be searched from inception to August 2025 to identify randomized controlled trials focusing on aromatherapy for CIGT management in patients with cancer. Data on participant characteristics, interventions, comparisons, outcomes, and adverse effects will be extracted from included studies. Continuous outcomes will be synthesized using standardized mean differences with 95% CIs, and categorical outcomes will be summarized as odds ratios with 95% CIs. All analyses will adopt a random-effects model to account for expected clinical and methodological heterogeneity. Subgroup and meta-regression analyses will be conducted to examine differences across essential oil types, intervention forms, and intervention durations. The Hartung-Knapp-Sidik-Jonkman method will be used for random-effects estimation, and prediction intervals will be calculated where applicable to reflect real-world variation. Risk of bias will be assessed using the Cochrane Risk of Bias 2 tool, and evidence certainty will be graded using the Grading of Recommendations Assessment, Development, and Evaluation (GRADE) approach.

**Results:**

This study was funded in February 2024. As of August 2025, the literature search and study selection have been completed, and 20 eligible randomized controlled trials have been identified. Data extraction and quantitative synthesis are expected to be completed in December 2025, and the final results are anticipated to be submitted for publication in March 2026.

**Conclusions:**

The anticipated findings will address key evidence gaps by evaluating aromatherapy’s therapeutic potential for CIGT beyond nausea and vomiting and clarifying parameter-specific effects on CIGT management. These findings will support the development of evidence-based, standardized aromatherapy interventions, guide future mechanism-based research, and inform clinical decision-making in supportive cancer care.

## Introduction

### Background

Cancer remains a major contributor to global morbidity and mortality, with an estimated 19.3 million new cases and nearly 10 million deaths reported in 2020 [1,2]. Chemotherapy is the primary treatment for cancer [3] and plays a crucial role in improving patient survival. However, it can induce a wide range of side effects, including myelosuppression, nephrotoxicity, neurotoxicity, gastrointestinal toxicity, alopecia, fatigue, and other systemic reactions [4,5]. Chemotherapy-induced gastrointestinal toxicity (CIGT) is the most commonly reported adverse effect [6]. It refers to the adverse effects on the gastrointestinal tract caused by chemotherapy drugs, manifesting as nausea, vomiting, appetite loss, oral mucositis, constipation, and diarrhea [7-9]. Previous studies have reported that nausea and vomiting affect 70% to 80% of patients [10], oral mucositis affects 40% to 75% [11], and diarrhea affects up to 80% of patients receiving chemotherapy [12]. These CIGT symptoms significantly compromise the quality of life of patients with cancer, increasing the risk of treatment nonadherence or discontinuation and even leading to death in case of severe gastrointestinal toxicity [13,14].

The current management of CIGT primarily relies on pharmacological interventions, which alleviate symptoms [5] but are hindered by their cost and often modest long-term efficacy, limiting their widespread clinical application. In recent years, complementary and alternative therapies have attracted increasing attention due to their efficacy in managing CIGT [5,15]. Aromatherapy, a type of complementary and alternative therapy, uses essential oils and other aromatic compounds derived from plants to enhance health and well-being [16]. It has empirical support for alleviating nausea, vomiting [17], oral mucositis, and constipation [18], which are key symptoms of CIGT.

Existing systematic reviews and meta-analyses have predominantly focused on aromatherapy’s effects on nausea and vomiting [17,18]. Notably, no systematic reviews have yet addressed aromatherapy’s role in managing other common CIGT symptoms, including appetite loss, oral mucositis, constipation, and diarrhea. These under-studied symptoms not only exhibit high incidence rates (comparable to nausea and vomiting in some patient cohorts [19,20]) but also share pathophysiological links with nausea and vomiting, and this co-occurrence often exacerbates patient distress and impairs treatment adherence [5]. Furthermore, critical gaps remain in understanding how aromatherapy parameters influence CIGT outcomes: variations in essential oil types (eg, peppermint, ginger, and lavender); intervention forms (eg, inhalation vs topical massage); and intervention durations (short term: <2 weeks; long term: ≥2 weeks) have not been systematically compared to determine their relative efficacy for CIGT. This lack of granularity limits the translation of existing evidence into standardized clinical practice.

Thus, this protocol aims to address these identified gaps in existing evidence: by systematically evaluating the efficacy of aromatherapy for the full spectrum of CIGT symptoms in patients with cancer and clarifying the effects of key aromatherapy parameters (essential oil types, intervention forms, and intervention durations) via subgroup analyses. By synthesizing comprehensive evidence on CIGT symptoms management and parameter-specific effects, this study will provide a foundation for developing evidence-based aromatherapy interventions and optimizing CIGT management strategies, ultimately supporting improved patient quality of life and treatment tolerance.

### Objectives

The primary objective of this systematic review and meta-analysis is to address the identified gaps in existing evidence regarding aromatherapy for CIGT in patients with cancer. Specifically, the study aims to achieve two core goals: (1) Systematically evaluate the efficacy of aromatherapy in alleviating the full spectrum of CIGT symptoms, including nausea, vomiting, appetite loss, oral mucositis, constipation, and diarrhea in patients with cancer, rather than focusing solely on nausea and vomiting, and (2) quantify the effects of key aromatherapy variables on CIGT outcomes through subgroup analyses, with a focus on 3 critical parameters: essential oil types (eg, peppermint, ginger, and lavender); intervention forms (eg, inhalation and topical massage); and intervention durations (eg, short term and long term). These goals will collectively clarify the clinical utility of aromatherapy for CIGT and provide parameter-specific references for clinical practice.

## Methods

### Registration

This study will follow the Preferred Reporting Items for Systematic Review and Meta-Analysis Protocols (PRISMA-P) guidelines [21] (Checklist 1). The protocol was officially registered in PROSPERO on August 28, 2024 (CRD42024578888).

### Design

This systematic review includes comprehensive meta-analyses based on aggregated data.

### Ethical Considerations

Ethics approval was not required for this study because it is a systematic review and meta-analysis that uses only aggregated data from previously published randomized controlled trials and does not involve the recruitment of human participants or access to identifiable individual-level data. According to our institutional ethics policies, secondary research based solely on published and fully deidentified data is exempt from research ethics board review.

### Eligibility Criteria

#### Types of Studies

This review will consider randomized controlled trials and include articles published in both English and Chinese, with no limitations on the year of publication. Studies with data that cannot be extracted or transformed, as well as duplicate publications, conference abstracts, reviews, editorials, letters, and protocols, will be excluded. The inclusion criteria were determined according to the participants, interventions, comparisons, outcomes, and study (PICOS) framework and guided by the Cochrane Handbook for Systematic Reviews of Interventions [22].

#### Types of Participants

Studies involving adult patients (>18 years) with a confirmed cancer diagnosis who were receiving chemotherapy will be included. There will be no restrictions on the type or stage of cancer or chemotherapy regimens.

#### Types of Interventions

The interventions to be assessed in this review will encompass all forms of aromatherapy regardless of dosage, method, or route of administration.

### Comparisons and Controls

Studies comparing aromatherapy with various control conditions, including blank controls, waitlist controls, placebos, usual care, and standard treatments, will be considered.

#### Types of Outcome Measures

The outcomes will include the following: (1) nausea, (2) vomiting, (3) diarrhea, (4) appetite loss, (5) constipation, (6) oral mucositis, (7) quality of life, (8) sleep quality, (9) anxiety, (10) depression, and (11) adverse events. Detailed assessment methods for each outcome are summarized in Table 1.

**Table 1.** Outcome assessment methods.

Outcomes	Assessment methods
Primary outcomes	
Nausea	Visual Analog Scale; Rhodes Index of Nausea, Vomiting and Retching; and various measures of intensity, duration, or other scales
Vomiting	Rhodes Index of Nausea, Vomiting and Retching, frequency measures, or other scales
Diarrhea	Severity grading scales or other validated tools
Appetite loss	Visual analog scale, changes in dietary intake, or other scales
Constipation	Constipation Assessment Scale or other scales
Oral mucositis	World Health Organization oral mucositis grading scale, Common Terminology Criteria for Adverse Events, or other scales
Secondary outcomes	
Quality of life	European Organization for Research and Treatment of Cancer, Quality of Life Questionnaire-Core 30, the Quality of Life Questionnaire-Lung Cancer 13, or other scales
Sleep quality	Pittsburgh Sleep Quality Index or other scales
Depression and anxiety	State-Trait Anxiety Inventory, Hospital Anxiety, and Depression Scale or other scales
Adverse events	Self-reported physical distress (eg, rash, malaise, dizziness, headache, or sneezing)

#### Searching Methods for the Identification of Studies

A comprehensive literature search will be conducted in PubMed, Cochrane Library, Web of Science, Embase, Cumulative Index to Nursing and Allied Health Literature, Chinese National Knowledge Infrastructure, Wanfang Database, Chinese Science and Technology Journal Database, and Chinese Biomedical Literature Service System from inception to August 2025. Additionally, the World Health Organization (WHO) trial portal, Chinese Clinical Trial Registry, gray literature, and reference lists of included studies will also be searched.

The search will be performed using Medical Subject Heading terms and free-text words related to cancer, aromatherapy, chemotherapy, and gastrointestinal toxicity. The detailed PubMed search strategy is presented in Table 2.

**Table 2.** PubMed search strategy.

Search no	MeSH terms and keywords	Items found
1	((((((neoplasms[MeSH Terms]) OR (tumor*[Title/Abstract])) OR (tumour*[Title/Abstract])) OR (neoplas*[Title/Abstract])) OR (cancer*[Title/Abstract])) OR (carcinoma*[Title/Abstract])) OR (malignan*[Title/Abstract])	5,334,288
2	(((((((((drug therapy[MeSH Terms]) OR (Chemoradiotherapy[MeSH Terms])) OR (Chemotherapy, Adjuvant[MeSH Terms])) OR (chemotherapy[Title/Abstract])) OR (pharmacotherapy[Title/Abstract])) OR (chemo*[Title/Abstract])) OR (chemical[Title/Abstract])) OR (chemotherapeutic[Title/Abstract])) OR (chemotherapeutant[Title/Abstract])) OR (chemical therapy[Title/Abstract])	2,960,959
3	(((((((((((((((aromatherapy[MeSH Terms]) OR (aroma*[Title/Abstract])) OR (essential oil*[Title/Abstract])) OR (fragrance[Title/Abstract])) OR (oils, volatile[MeSH Terms])) OR (volatile oil*[Title/Abstract])) OR (aroma therap*[Title/Abstract])) OR (aromatic massage[Title/Abstract])) OR (inhalational aroma[Title/Abstract])) OR (inhalation aromatherapy[Title/Abstract])) OR (massage aromatherapy[Title/Abstract])) OR (incense therap*[Title/Abstract])) OR (natural medicine[Title/Abstract])) OR (Medicine, Traditional[MeSH Terms])) OR (phytotherapy[MeSH Terms])) OR (plants, medicinal[MeSH Terms])	331,508
4	(((((((((((randomized controlled trial[MeSH Terms]) OR (Double-blind method[MeSH Terms])) OR (Single-blind method[MeSH Terms])) OR (controlled clinical trial[Publication Type])) OR (Clinical Trial[Publication Type])) OR (RCT[Title/Abstract])) OR (random*[Title/Abstract])) OR (Random Allocation[MeSH Terms])) OR (alloc*[Title/Abstract])) OR (assign*[Title/Abstract])) OR (placebo[Title/Abstract])) OR (trial[Title/Abstract])	2,535,445
5	#1 AND #2 AND #3 AND #4	1855

### Data Collection

#### Selection of Studies

All references retrieved from the 9 databases and 2 trial registries will be imported into Endnote X9 (Clarivate), and duplicate entries will be eliminated. Two reviewers will independently screen the titles and abstracts to identify studies eligible for inclusion. Secondary screening will involve a detailed examination of the full texts to determine eligibility according to the selection criteria. Any disagreements between the reviewers will be resolved by consensus. If the 2 reviewers are unable to reach a consensus, a third reviewer will be sought to make a final decision.

#### Data Extraction and Management

Two reviewers will perform independent data extraction from the eligible studies, and the data will be entered into Microsoft Excel 2016. The data will include study characteristics (first author, country, and publication year), characteristics of participants (sample size, age, sex, cancer type, and chemotherapy regimens), details of the intervention and comparison (dose and route of administration), outcomes, adverse effects, and conclusions. To minimize reporting bias, we will request missing data, including unreported outcomes or adverse events, from corresponding authors of published trials or investigators identified through trial registries via email once any missingness is identified during data extraction. Data will be extracted by 2 independent reviewers and any differences will be promptly resolved by consensus.

#### Assessment of Risk of Bias

The quality of the studies will be independently evaluated by 2 reviewers using the Cochrane tool for assessing the risk of bias in randomized trials version 2 (RoB2) [23]. The RoB2 encompasses 5 domains and 22 items, with response categories including “yes,” “probably yes,” “probably no,” “no,” and “no information.” Each item is assessed as “low risk of bias,” “some concerns,” or “high risk of bias.” A consensus will be reached through a meeting between the 2 reviewers. If disagreements remain between the two initial reviewers after discussion, a third reviewer will be consulted; if consensus is still not reached, final decisions will be made through discussion within the review team.

#### Data Analysis

The meta-analysis will be performed using RevMan software (version 5.2; The Cochrane Collaboration). Continuous data will be synthesized using standardized mean differences and 95% CIs to account for differences in measurement tools across studies, whereas categorical data will be evaluated using odds ratios and 95% CIs to quantify associations between aromatherapy and CIGT outcomes. All meta-analyses will be conducted using a random-effects model to account for potential clinical and methodological heterogeneity across studies [24]. The heterogeneity of the studies will be evaluated using the *Q* test and the *I*^2^ statistic, and 95% prediction intervals, where applicable, will be calculated to indicate the range of true effects across studies. The Hartung-Knapp-Sidik-Jonkman [25] method will be applied for random-effects estimation to improve the precision and robustness of pooled effect sizes. Potential outliers will be graphically assessed using forest plots. Subgroup analyses will be prespecified to assess potential sources of clinical heterogeneity, including essential oil types (eg, ginger, peppermint, and lavender); intervention forms (eg, inhalation and topical massage); and intervention durations (eg, short term and long term). If sufficient studies are available, meta-regression analysis will be performed to further explore potential sources of heterogeneity. Sensitivity analyses will be conducted to assess the stability and reliability of the pooled estimates. Additionally, when substantial heterogeneity remains unexplained, results will be supplemented with a narrative synthesis.

#### Publication Bias Assessment

Small-study effects will be evaluated as an indirect approach to explore potential publication bias. Funnel plots will be visually inspected, and the Egger test (*P*<.05) will be performed if ≥10 studies are included in the meta-analysis. If substantial small-study effects are detected, the trim-and-fill method will be applied to assess the potential impact on the robustness of pooled results. Funnel plot asymmetry tests will not be performed if the SEs of intervention effect estimates are similar (ie, studies are of comparable size), as such tests lack statistical power in this context.

#### Grading the Quality of Evidence

The Grading of Recommendations Assessment, Development, and Evaluation (GRADE) methodology [26] will be used to assess the quality of evidence for all outcomes. The quality of evidence will be graded as “high,” “moderate,” “low,” and “very low,” based on domains including risk of bias, inconsistency, indirectness, imprecision, and publication bias. Inconsistency will be judged according to the degree of heterogeneity (*I*² statistic and overlap of CIs), while imprecision will be evaluated based on the width of CIs and the total sample size relative to the optimal information size. Two reviewers will perform the GRADE assessment independently. If disagreements remain after discussion, a third reviewer will be consulted; if consensus is still not reached, final decisions will be made through discussion within the review team.

#### Dissemination

The findings will be disseminated via publication in peer-reviewed journals and presentations at academic conferences.

## Results

**Figure 1.**
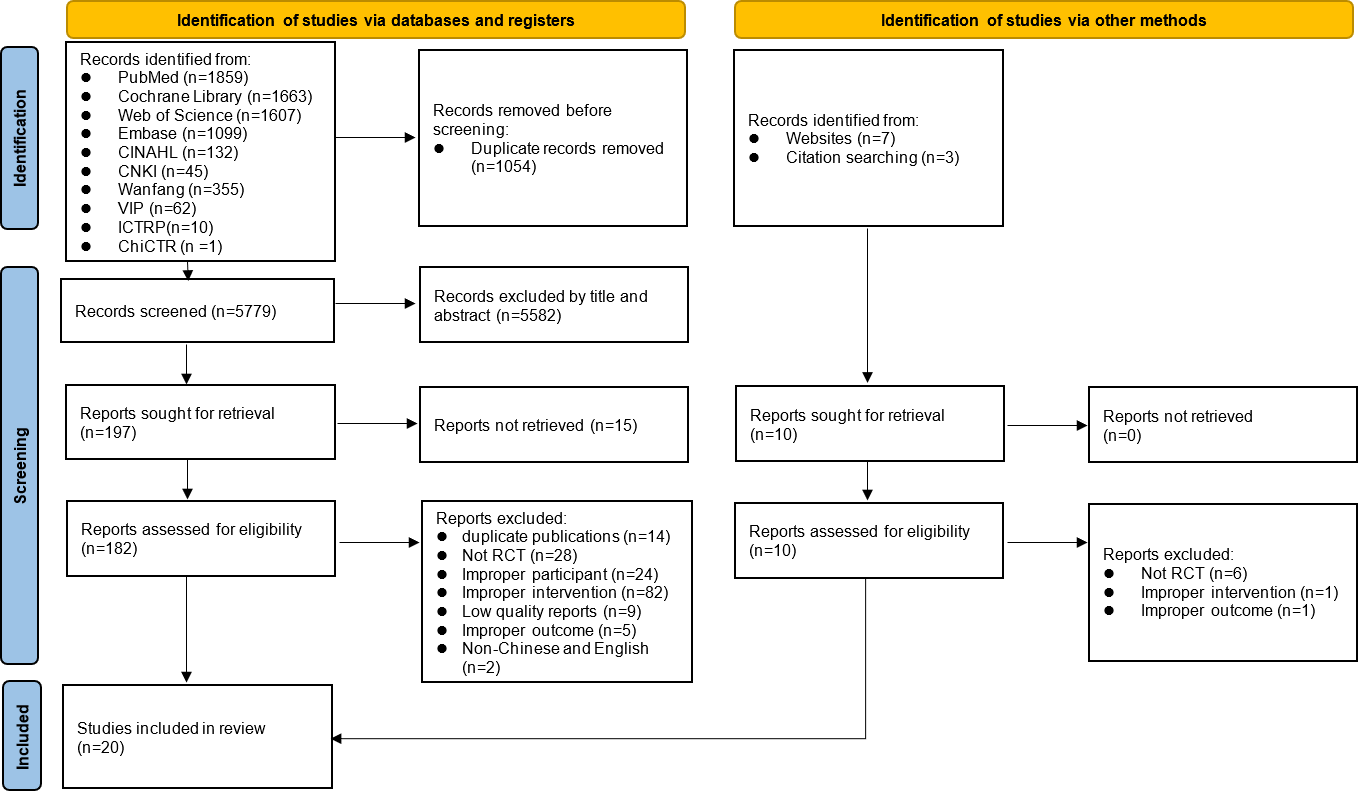
Study flow diagram. ChiCTR: Chinese Clinical Trial Registry; CINAHL: Cumulative Index to Nursing and Allied Health Literature; CNKI: Chinese National Knowledge Infrastructure; ICTRP: International Clinical Trials Registry Platform; RCT: randomized controlled trial; VIP: Chinese Science and Technology Journal Database.

The literature search was completed in August 2025, identifying 6833 records from 9 databases and 2 trial registries and an additional 10 records from other sources (websites, n=7; citation searching, n=3), for a total of 6843 records. Among the records from databases and trial registries, 1054 duplicate entries (15.4%) were removed, leaving 5779 records for title and abstract screening. In total, 184 full-text articles (2.7% of all identified records), including 182 records from databases and trial registries and 2 from other sources, were assessed for eligibility. Ultimately, 20 randomized controlled trials (0.3%) met the predefined eligibility criteria (Figure 1). Quality assessment and data synthesis are currently underway and are expected to be completed by December 2025. The final results of the review are anticipated to be available in March 2026. Given that this review synthesizes data from existing published studies, no direct participant recruitment is required.

## Discussion

### Anticipated Findings

This systematic review and meta-analysis aims to summarize current evidence on aromatherapy for CIGT and specifically fill key gaps in existing research, by clarifying aromatherapy’s effects on the full spectrum of CIGT symptoms (ie, nausea, vomiting, appetite loss, constipation, diarrhea, and oral mucositis) rather than focusing solely on nausea and vomiting. All analytical procedures will adhere to PRISMA and Cochrane Collaboration standards to ensure methodological rigor, transparency, and reproducibility. The methodological quality of included studies will be independently assessed by 2 reviewers using the RoB2 tool, and the certainty of evidence will be appraised via the GRADE approach.

To explore differential therapeutic effects, we will systematically analyze differences in essential oil types, intervention forms, and durations. Subgroup analyses will be conducted to examine potential variations associated with essential oil types (eg, ginger, peppermint, and lavender); intervention forms (eg, inhalation and topical massage); and intervention durations (eg, short term: <2 weeks; long term: ≥2 weeks) based on clinical practice. These analyses are expected to clarify how intervention parameter differences influence outcomes, addressing the lack of granularity in parameter-related evidence.

By conducting a rigorous literature search, structured data extraction, and the aforementioned quality appraisal, this review will deliver a comprehensive synthesis of existing evidence. Specifically, it will clarify aromatherapy’s overall effectiveness in managing CIGT, the consistency of its effects across different symptoms, and the clinical applicability of specific aromatherapy regimens.

Ultimately, the findings may lay 3 key foundations: first, to inform the development of evidence-based aromatherapy interventions for CIGT, thereby addressing the limitation of “nonstandardized practice” in existing research; second, to optimize supportive care for patients with cancer by reducing CIGT-related distress, improving quality of life, and enhancing treatment adherence, which directly addresses the clinical harms of CIGT highlighted in the Introduction; and third, to guide future research exploring the underlying mechanisms by which aromatherapy alleviates CIGT, thus promoting the development of mechanism-driven, more targeted interventions.

### Strengths and Limitations

This systematic review has several methodological strengths: it adheres to PRISMA-P and Cochrane Collaboration standards to ensure rigor and transparency; 2 reviewers will independently conduct literature selection, data extraction, and bias assessment, with discrepancies resolved by consulting a third reviewer; the RoB2 tool and GRADE approach will be used to enhance the reliability of results; and subgroup analyses will explore heterogeneity related to essential oil types, intervention forms, and intervention durations.

Nonetheless, certain limitations should be acknowledged: restricting the search to English and Chinese studies may introduce language bias; heterogeneity in aromatherapy interventions (eg, oil concentration and dosage regimens) could impact pooled results; unpublished negative studies may lead to publication bias; and high methodological heterogeneity among included studies may weaken the credibility of conclusions.

Despite these limitations, the findings are expected to serve as a scientific foundation for developing standardized clinical guidelines, which will ensure the safe, effective, and evidence-based integration of aromatherapy into oncology care. Ultimately, this will enable health care professionals to select appropriate essential oils, determine optimal dosages, and apply suitable administration methods, thus improving patient outcomes and promoting high-quality, patient-centered supportive care [27]. The findings will also be disseminated through peer-reviewed publications and academic conference presentations to facilitate the translation of evidence into clinical oncology practice.

### Conclusions

This protocol outlines a comprehensive systematic review and meta-analysis aimed at evaluating the efficacy and safety of aromatherapy for CIGT in patients with cancer. Evidence from randomized controlled trials will be synthesized to clarify the therapeutic value of aromatherapy; identify effective essential oil types, intervention forms, and intervention durations; and resolve inconsistencies in existing evidence. The anticipated results will provide an evidence-based foundation for the safe and standardized integration of aromatherapy into supportive cancer care, while guiding future research on mechanistic exploration, optimized intervention protocols, and long-term clinical evaluations.

## Supplementary material

10.2196/76350 Checklist 1 PRISMA-P checklist.

## Abbreviations

CIGT: chemotherapy-induced gastrointestinal toxicity
GRADE: Grading of Recommendations Assessment, Development, and Evaluation
PICOS: participants, interventions, comparisons, outcomes, and study
PRISMA-P: Preferred Reporting Items for Systematic Review and Meta-Analysis Protocols
RoB2: Risk of Bias 2
WHO: World Health Organization
